# Mediterranean Diet Adherence, Physical Activity, and Motivation Toward Physical Education in Adolescent Girls: A Cross-Sectional Study

**DOI:** 10.3390/healthcare14060764

**Published:** 2026-03-18

**Authors:** Paula San Martín González, Natalia Hermida Carballido, Rubén Maneiro Dios, Rubén Arroyo del Bosque

**Affiliations:** 1 Department of Nursing, Faculty of Health Sciences, Pontifical University of Salamanca, 37002 Salamanca, Spain; psanmartingo@upsa.es; 2 Department of Health Sciences, Faculty of Health Sciences, University of Burgos, 09001 Burgos, Spain; nhermida@ubu.es; 3 Department of Special Didactics, Faculty of Education and Sports Sciences, University of Vigo, 36005 Pontevedra, Spain; ruben.maneiro@uvigo.gal; 4 Department of Specific Didactics, Faculty of Education, University of Burgos, 09001 Burgos, Spain

**Keywords:** healthy lifestyle behaviours, health promotion, self-determination theory, school health programs, Mediterranean diet, adolescent girls

## Abstract

**Highlights:**

**What are the main findings?**

- Greater adherence to the Mediterranean diet was associated with higher levels of self-determined motivation toward physical education among adolescent girls.
- Physically active girls showed higher intrinsic motivation toward physical education compared with their less active peers.

**What are the implications of the main findings?**

- Similar patterns across urban and rural environments suggest that school-based interventions promoting healthy lifestyles may be broadly applicable.
- Integrating strategies that promote healthy eating and physical activity within school settings may strengthen motivation toward physical education among adolescent girls.

**Abstract:**

**Background:** Adolescence represents a critical period for the adoption of lifestyle behaviors that may influence physical health, emotional well-being, and health-related behaviors later in life. However, limited evidence exists regarding the combined association of dietary habits and physical activity with motivation toward physical education, particularly among adolescent girls from different residential environments. **Objective:** This study aimed to examine the relationship between adherence to the Mediterranean diet, physical activity levels, and motivation toward physical education among adolescent girls from urban and rural settings. **Methods:** A cross-sectional study was carried out involving girls aged 12 to 14 years (n = 217; N_Urban_ = 108 and N_Rural_ = 109). Adherence to the Mediterranean diet, physical activity levels, and motivational dimensions toward PE were assessed using validated questionnaires. Differences between groups were analyzed using analysis of variance (ANOVA), and an analysis of covariance (ANCOVA) was performed controlling for physical activity levels. Effect sizes were calculated using partial eta squared (η^2^p). **Results:** Significant differences were observed in intrinsic motivation, identified regulation, introjected regulation, and amotivation according to adherence to the Mediterranean diet (*p* < 0.05), with small to moderate effect sizes (η^2^p = 0.029–0.040). Post hoc analyses indicated that girls with optimal adherence to the Mediterranean diet exhibited higher intrinsic motivation toward PE compared with those with low adherence. The ANCOVA revealed that higher physical activity levels were significantly associated with greater intrinsic motivation, particularly among girls from urban environments. No significant differences were found between urban and rural environments in overall physical activity levels or dietary adherence. **Conclusions:** Greater adherence to the Mediterranean diet and higher levels of physical activity are associated with more self-determined motivational profiles toward physical education in adolescent girls. These findings highlight the importance of integrated school-based interventions that promote healthy eating and active lifestyles to enhance motivation and engagement in PE among adolescent girls.

## 1. Introduction

Adolescence is a key transitional period marked by rapid biological maturation and psychosocial development, during which health-related behaviors that may influence well-being across the life course are established [1,2,3]. Among these behaviors, regular participation in physical activity (PA) and adherence to healthy dietary patterns have been consistently linked to a reduced risk of chronic conditions and to the promotion of health across the life span [4,5,6].

In recent years, several studies have shown an increase in unhealthy behaviours among the adolescent population, mainly associated with increased sedentary behaviour, excessive use of technological devices, and the adoption of unhealthy dietary habits, factors that have contributed to the rise in overweight and obesity [7,8,9].

This critical stage of development is characterized by profound and multi-dimensional transformations that are particularly evident in females. From a physical and morpho-functional perspective, adolescent girls undergo significant changes in body composition, primarily driven by hormonal fluctuations and the increase in estrogens [10]. These physiological shifts result in a relative increase in fat mass and the maturation of the skeletal system, which is essential for the acquisition of peak bone mass [11]. However, these changes can also lead to a temporary decline in motor efficiency and a negative perception of physical self-concept, which are often associated with a decrease in regular PA levels during this stage. From a psychological perspective, this period is marked by the development of formal operational thought and a complex process of identity construction. Adolescents experience increased emotional vulnerability and a heightened sensitivity to social evaluation and peer influence [12]. In the case of females, studies have reported a more pronounced decline in self-esteem and body satisfaction compared to males, directly impacting their motivation toward physical-sporting activities [13]. Furthermore, the maturation of the prefrontal cortex—the area responsible for executive functions and self-regulation—is still ongoing during early adolescence [14]. This neurobiological development explains why external support and the educational environment are decisive in the internalization of healthy habits, such as adherence to the Mediterranean diet (MD) and the maintenance of active lifestyles. The synergy between these biological, functional, and psychological changes justifies the need for an integrated approach to understand how nutritional patterns and motivational processes in Physical Education (PE) can act as protective factors for health during this vulnerable stage.

Moreover, despite the well-documented benefits of MD, a progressive decline in adherence to this dietary pattern has been observed during adolescence, which may have negative consequences both at this stage and in adulthood [13,15]. Scientific evidence indicates that the combination of a balanced diet and regular PA is essential for maintaining an adequate health status and a healthy weight status [16,17].

The analysis of these habits in adolescent girls is of particular relevance, as social, cultural, and environmental factors may specifically influence their PA levels, adherence to healthy dietary patterns, and motivation towards engagement in PA and sport [18,19].

The decision to focus exclusively on adolescent girls responds to consistent evidence indicating that girls report lower levels of PA, higher dropout rates from organized sport, and greater declines in motivational engagement during early adolescence compared with boys. These differences are shaped by both internal factors, such as low motivation, negative self-perception, and pubertal changes, and external factors, including gender stereotypes, lack of female role models, and non-inclusive environments [20]. Gender-specific sociocultural pressures, body image concerns, and differential perceptions of competence in PE, where girls often perceive lower competence and greater body dissatisfaction, may uniquely influence their motivational experiences and lifestyle behaviors [20,21]. Consequently, examining these relationships within an exclusively female sample allows for a more precise understanding of the factors shaping health-related behaviors and motivational profiles in this population.

In addition, residential environment (rural or urban) may shape access to sports facilities, opportunities for active commuting, and daily routines, potentially contributing to differences in lifestyle patterns among adolescents [7,13,22]. Understanding how contextual and behavioral factors interact is crucial for designing effective, context-sensitive health promotion strategies.

Likewise, motivation toward PE classes has been identified as a key determinant of sustained engagement in PA, as higher motivation is associated with greater adherence to active and healthy lifestyles [23,24,25]. Identifying the factors that shape this motivation is therefore essential for the design of effective educational and health promotion interventions.

From a theoretical perspective, students’ motivation toward PE can be understood within the framework of Self-Determination Theory (SDT) [26]. SDT conceptualizes motivation along a continuum ranging from intrinsic motivation, engaging in an activity for inherent enjoyment and interest, to different forms of extrinsic motivation (identified, introjected, and external regulation), and ultimately to amotivation, characterized by a lack of intention or perceived value in the activity. According to this theory, more self-determined forms of motivation are associated with greater engagement, persistence, and psychological well-being, whereas controlled regulation and amotivation are linked to lower participation and potential disengagement from PA during adolescence [27].

Within this theoretical framework, lifestyle behaviors may not only represent outcomes of motivational processes but also contextual factors that shape the quality of motivation itself.

Although the relationship between PA and motivational processes has been widely examined, the potential association between dietary patterns and motivation toward PE remains less explored. Adherence to the MD, characterized by high consumption of fruits, vegetables, whole grains, legumes, and healthy fats, has been associated not only with improved physical health but also with enhanced psychological well-being and mood regulation in adolescent populations. From a biopsychosocial perspective, adequate nutritional intake may contribute to greater perceived vitality, improved emotional regulation, and sustained energy levels, which could indirectly support more autonomous forms of motivation toward PA and participation in PE classes. However, the theoretical mechanisms linking dietary habits and motivational profiles remain insufficiently clarified, particularly among adolescent girls.

Although previous research has explored associations between MD adherence, PA, and motivational variables in adolescent populations [28], important gaps remain. Prior studies have primarily examined mixed-gender samples without specifically addressing gender-focused differences or contextual residential factors.

Furthermore, limited evidence has analyzed these associations exclusively in adolescent girls while simultaneously adopting a gender-focused and context-sensitive approach grounded in SDT. Therefore, the present study extends previous findings by focusing specifically on female adolescents and by examining potential contextual influences related to place of residence.

Accordingly, grounded in SDT, this study aimed to examine whether adherence to the MD and PA levels is associated with distinct motivational orientations toward PE among adolescent girls, considering potential differences between rural and urban contexts, in order to provide evidence for context-sensitive strategies to promote healthy lifestyles within the school setting.

## 2. Materials and Methods

### 2.1. Participants

This research employed a quantitative approach using a cross-sectional descriptive–correlational design [29], with the objective of analyzing the relationship between adherence to MD and motivation towards PA among Spanish adolescent girls, considering the residential environment (urban or rural).

Data were collected during the 2024–2025 academic cycle in public and publicly funded educational centers located in both rural and urban areas of Salamanca (Spain). Residential environment (urban vs. rural) was classified according to the official population criteria established by the Spanish National Statistics Institute. Municipalities with fewer than 10,000 inhabitants were categorized as rural, whereas those with 10,000 or more inhabitants were classified as urban.

The study sample consisted of adolescents who met the following inclusion criteria: being enrolled in one of the participating schools, being between 12 and 14 years old, providing written informed consent from their legal guardians, and regularly attending PE classes with a willingness to complete the questionnaires.

Participants who reported having chronic diseases, musculoskeletal disorders, or medical restrictions that prevented regular participation in physical activities were not included, nor were those whose questionnaires were incomplete or contained clearly inconsistent responses.

An initial sample of 232 adolescents was recruited. After applying the inclusion and exclusion criteria, the final sample consisted of 217 participants, of whom 108 were from urban environments and 109 from rural environments, all aged between 12 and 14 years.

A non-probabilistic convenience sampling strategy was employed, based on the accessibility of the educational centres and the feasibility of conducting data collection within the available timeframe.

Although this strategy facilitates participation and optimizes available resources, it limits the generalizability of the results to broader populations. To minimize potential selection bias, schools with diverse sociodemographic characteristics were included, and efforts were made to maintain a balanced representation according to rural and urban environments, as well as participants’ PA levels.

### 2.2. Instruments

Data were collected using the following validated questionnaires:

First, an ad hoc sociodemographic questionnaire was implemented to collect information on age, residential environment, and PA habits.

Participants’ PA levels were assessed using the International Physical Activity Questionnaire for Adolescents (IPAQ-A) [30], an instrument specifically designed for the adolescent population and with adequate validity and reliability. The questionnaire consists of 11 items, organized into four sections: PA at school, household and gardening activities, active transportation, and PA during leisure time. Specifically, the PA patterns assessed in this adolescent population include structured activities such as school-based PE and extracurricular sports (e.g., volleyball, dance, or gymnastics), as well as unstructured PA like active commuting to school (walking or cycling) and recreational play. These activities are categorized by intensity to ensure they align with the aerobic and muscle-strengthening efforts recommended for functional development during adolescence. The items collect information on the frequency and duration of PA performed over the previous seven days, differentiated by activity intensity, and include one item assessing sedentary behaviours. The IPAQ-A considers three intensity levels (light, moderate, and vigorous), distributed across three items on light PA, four on moderate PA, and three on vigorous PA, allowing for a detailed characterization of PA patterns in both school and extracurricular contexts. Based on the results, participants were classified into low, medium, or high PA levels according to compliance with PA recommendations for adolescents, thereby facilitating data presentation and analysis.

Adherence to the MD was evaluated using the KIDMED index [31,32], a validated questionnaire frequently employed in studies involving Spanish adolescent populations [13,24,33,34]. The MD pattern for this age group is characterized by a high intake of vitamins, minerals, and healthy fats necessary for pubertal growth. It prioritizes the daily consumption of fruits, vegetables, and cereals, the use of olive oil as the primary lipid source, and a consistent intake of dairy products for bone health. Furthermore, it encourages the consumption of pulses and fish while limiting the intake of ultra-processed foods and industrial sugars, which is crucial for maintaining metabolic balance during the adolescent transition. The instrument consists of 16 dichotomous items (yes/no) that assess dietary habits related to the Mediterranean dietary pattern. Positive dietary behaviors are assigned one point, whereas negative behaviors are penalized with one point. Based on the overall score, participants were categorized into three levels of adherence: high adherence (≥8 points), intermediate adherence (4–7 points), and low adherence (≤3 points).

Motivation toward PE was assessed using the Physical Education Motivation Questionnaire (PEMQ) [35]. This instrument comprises 20 items introduced by the statement “I participate in Physical Education classes…” and evaluates five motivational dimensions. Internal consistency analyses indicated acceptable reliability across all factors, with Cronbach’s alpha values ranging from 0.77 to 0.83. The dimensions assessed were intrinsic motivation, identified regulation, introjected regulation, external regulation, and amotivation.

### 2.3. Procedure

Prior to data collection, ethical approval was obtained in compliance with the Declaration of Helsinki and its later amendments [36]. The study protocol was reviewed and approved by the Ethics Committee at the University of Burgos (approval code: 2024/REGSED-2113/ Nº IO 18/24) in accordance with the ethical principles established in the Declaration of Helsinki (1964) and its subsequent amendments [36]. Likewise, the recommendations of the American Psychological Association [37] regarding research with underage populations were followed.

To ensure consistency in data collection, a standardized protocol was implemented to promote uniform participation across the sample. Before data collection began, the schools involved were approached by the research team to provide detailed information about the study aims and the procedures to be followed.

After obtaining authorization from the participating secondary education centres, teachers were provided with a detailed explanation of the objectives and characteristics of the study. Subsequently, an informed consent document was provided to parents or legal guardians, which was a mandatory requirement for the participation of underage students. The document described the aims of the research, the conditions of confidentiality in data handling, and the participants’ right to withdraw from the study at any time without any consequences.

After informed consent was obtained, participants received a brief explanation of the study objectives, and the confidentiality and anonymity of all collected data were ensured. Data collection was conducted in the classroom setting, with a member of the research team present throughout the process to resolve any questions that could arise. The administration of the questionnaires required approximately 10–15 min and was carried out under standardized conditions to ensure a consistent and controlled environment for all participants.

### 2.4. Statistical Analysis

Prior to conducting the main statistical analyses, the distribution of the variables was examined to verify the assumptions of normality and homogeneity of variance required for parametric testing. Normality was assessed using the Kolmogorov–Smirnov test (n = 217), which indicated no significant deviations from normality. Homogeneity of variance was confirmed using Levene’s test.

To examine potential differences between groups across the motivational dimensions, a multivariate analysis of variance (MANOVA) was initially conducted to evaluate the overall effect of the independent variables on the set of dependent variables simultaneously. This approach allowed the simultaneous examination of multiple related motivational outcomes, thereby reducing the risk of inflated Type I error associated with conducting several independent tests. When significant multivariate effects were detected, follow-up analyses of variance (ANOVA) and analyses of covariance (ANCOVA) were performed to identify specific group differences. In the ANCOVA models, adherence to the MD and PA levels was included as fixed factors, while residential environment (rural vs. urban) was introduced as an additional factor in the model.

To address the potential inflation of Type I error due to multiple comparisons across the five motivational dimensions of the Physical Education Motivation Questionnaire (PEMQ), Bonferroni post hoc adjustments were applied to all pairwise comparisons. Effect sizes were calculated using partial eta-squared (η^2^p) and interpreted according to conventional benchmarks (small ≈ 0.01, medium ≈ 0.06, large ≈ 0.14). Statistical significance was set at *p* < 0.05. All statistical analyses were performed using SPSS version 28.0 (IBM Corp., Armonk, NY, USA).

## 3. Results

Before the main analyses, we considered whether the data were appropriate for parametric testing. According to the Kolmogorov–Smirnov test, the variables did not deviate significantly from a normal distribution (*p* > 0.05). In addition, Levene’s test was used to verify the homogeneity of variances, and this showed no relevant differences between the groups (*p* > 0.05), which supports the use of parametric statistical procedures.

The distribution of PA levels and MD adherence according to residential environment is presented in Table 1. No statistically significant differences were observed between adolescent girls from urban and rural environments in either PA levels (χ^2^ = 0.35; *p* = 0.705) or MD adherence (*p* = 0.940). In both groups, most participants were classified at moderate PA levels (25.8% in urban environments and 27.2% in rural environments), with a slightly higher trend among rural adolescent girls, although this did not reach statistical significance, followed by the low level (16.6% and 17.5%, respectively). These results indicate that the residential environment is not associated with significant differences in PA habits or dietary patterns in the analyzed sample.

### Motivation Towards Physical Education According to Mediterranean Diet Adherence

Analysis of variance between groups showed significant differences in several motivational dimensions toward PE according to adherence to the MD (Table 2). Specifically, significant differences were observed in intrinsic motivation (F = 4.14; *p* = 0.017; η^2^p = 0.037), with adolescents showing optimal MD adherence scoring higher than those with low adherence (O vs. L: *p* = 0.012; I vs. L: not significant). Similarly, significant differences were found in identified regulation (F = 3.84; *p* = 0.023; η^2^p = 0.035), with higher scores among adolescents with optimal MD adherence compared with low adherence (O vs. L: *p* = 0.018; I vs. L: not significant). Significant differences were also observed in introjected regulation (F = 4.42; *p* = 0.013; η^2^p = 0.040), again with higher values among adolescents with optimal adherence compared with low adherence (O vs. L: *p* = 0.020; I vs. L: not significant). In contrast, significant differences were found in amotivation (F = 3.12; *p* = 0.046; η^2^p = 0.029), with higher scores among adolescents with low MD adherence compared with optimal adherence (L vs. O: *p* = 0.039; L vs. I: not significant). No statistically significant differences were observed in external regulation (F = 0.43; *p* = 0.653; η^2^p = 0.002). Post hoc analyses indicate that greater adherence to the MD is associated with more self-determined motivational profiles, whereas lower adherence is associated with higher levels of amotivation.

**Figure 1:**
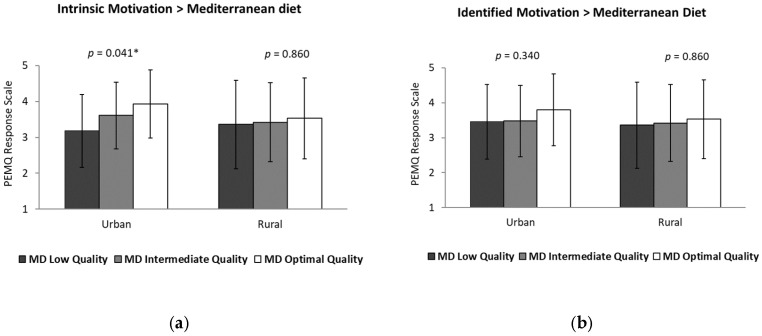

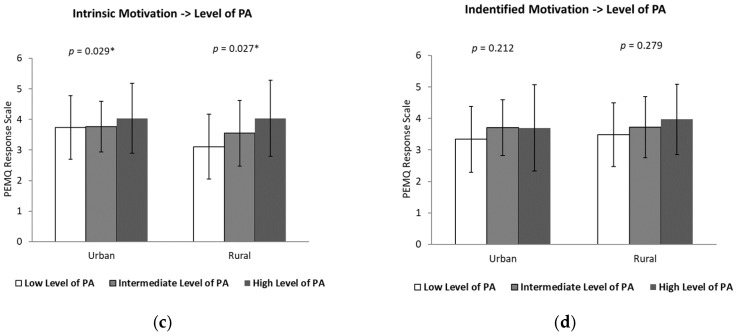
(**a**,**b**) Association between intrinsic and identified motivation toward physical education according to adherence to the Mediterranean diet (low, intermediate and optimal) and (**c**,**d**) physical activity level (inactive vs. active) in adolescent girls. * = *p* < 0.005.

The analysis of covariance (ANCOVA) showed statistically significant differences in intrinsic motivation towards PE according to the level of MD adherence, particularly among adolescents from urban environments (*p* < 0.05). Specifically, adolescents with optimal MD adherence showed significantly higher intrinsic motivation scores compared with those with low adherence (*p* < 0.05), in both urban and rural environments. Likewise, PA level was significantly associated with higher levels of intrinsic motivation (*p* < 0.05), regardless of the residential environment (Figure 1).

**Figure 2:**
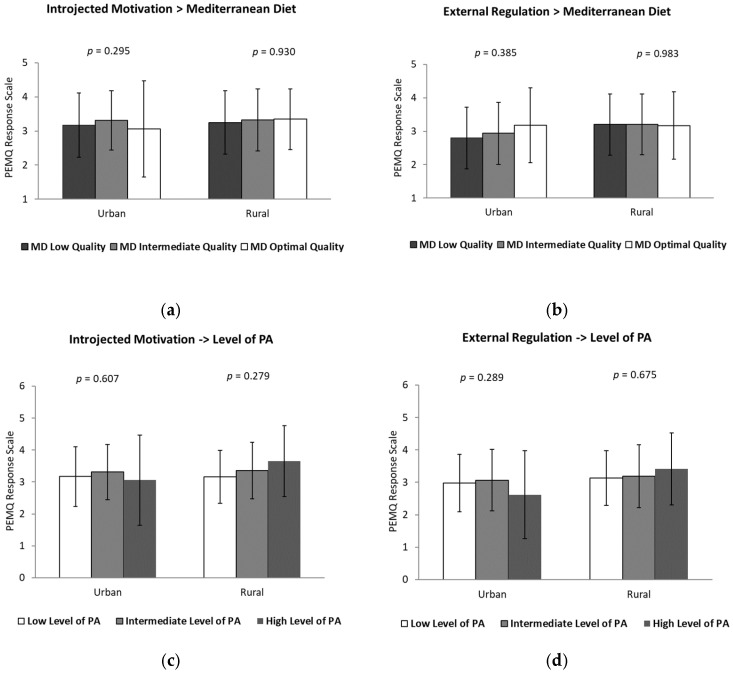
Introjected and external regulation toward physical education according to adherence to the Mediterranean diet (**a**,**b**) and physical activity level (**c**,**d**) in adolescent girls from urban and rural environments.

**Figure 3:**
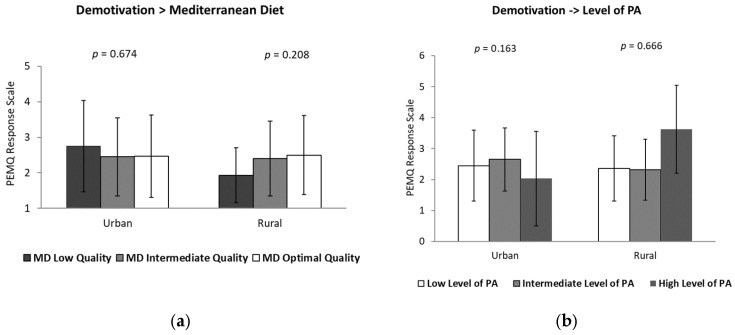
Amotivation toward physical education according to adherence to the Mediterranean diet (**a**) and physical activity level (**b**) in adolescent girls from urban and rural environments.

Subsequently, the results for the remaining dimensions are presented in Figure 2 and Figure 3.

Introjected and external regulation toward PE according to adherence to the MD and PA level across residential environments are presented in Figure 2. Visual inspection of the figure suggests slight variations in mean scores across categories of MD adherence and PA levels. Although the ANOVA results indicated significant differences in introjected regulation according to MD adherence, the magnitude of these differences was small. In both urban and rural environments, adolescents with low, medium, or high adherence to the MD showed relatively similar levels of introjected and external regulation. Likewise, motivational scores related to these regulatory styles did not differ significantly according to PA levels within either residential context. Overall, external regulation did not show statistically significant differences according to MD adherence or PA level among adolescent girls, regardless of their residential environment.

Amotivation toward PE according to adherence to the MD and PA level across residential environments is presented in Figure 3. Specifically, amotivation scores were comparable among adolescents with low, medium, and high adherence to the MD in both urban and rural environments. Similarly, no statistically significant differences were found in amotivation according to PA levels across residential contexts. These findings indicate that, despite minor fluctuations in mean scores, neither dietary adherence nor PA level appears to be associated with meaningful differences in amotivation toward PE among adolescent girls, regardless of whether they live in urban or rural settings.

## 4. Discussion

The main objective of the present study was to analyze the relationship between MD adherence, PA level, and motivation towards PE among Spanish adolescent girls, also considering residential environment. This approach is supported by previous evidence highlighting the interrelationship between healthy dietary habits, PA, and motivational variables in adolescent populations [38,39]. The findings indicate that higher MD adherence is associated with more self-determined motivational profiles towards PE.

One of the main findings of the study is that adolescents with optimal MD adherence exhibit significantly higher levels of intrinsic motivation, identified regulation, and introjected regulation towards PE classes compared with those with low adherence. These results are consistent with recent research that has reported positive associations between MD adherence and more self-determined motivational profiles in the context of PE [28], as well as with reviews highlighting the role of autonomous motivation in the adoption of active lifestyles among children and adolescents [27]. These results are consistent with the framework of Self-Determination Theory, which posits that healthy lifestyles are associated with greater autonomous regulation of behaviour.

MD, characterized by a high consumption of fruits, vegetables, legumes, and healthy fats, has previously been associated with better psychological and emotional well-being in adolescent populations, including higher levels of self-esteem and life satisfaction [32,40]. In this sense, our results suggest that a healthier diet may promote a more positive and autonomous disposition towards PA in the school context. These benefits can be explained not only from a psychological perspective, but also through physiological mechanisms related to diet quality.

The findings of this research are consistent with previous results indicating that healthier eating patterns are associated with better psychological outcomes and more active participation in physical exercise [38,41]. According to previous research, certain nutritional elements of the MD may contribute to improved perceived energy levels and mood help optimize energy levels and mood, which could be a possible reason why a relationship has been noted between diet quality and more self-determined motivational profiles [42].

Similarly, the literature has pointed out that this eating pattern has an impact not only on metabolic health, but also on other elements such as sleep, muscle recovery, and emotional well-being; the latter may have an indirect influence on motivation toward PA [43,44].

In addition, the analysis of covariance revealed that PA level was significantly associated with intrinsic motivation towards PE, independently of MD adherence level. This finding is consistent with previous studies showing that more physically active adolescents exhibit higher levels of self-determined motivation and greater engagement in PE classes [45,46]. This finding reinforces the idea that regular PA not only provides physical benefits but also motivational ones, fostering more self-determined forms of engagement in the subject.

The fact that the relationship between MD and motivation remains after controlling for PA suggests that both healthy behaviours may act in a complementary manner, jointly contributing to a more adaptive motivational profile.

Consistent with the previous findings, adolescents with low MD adherence exhibited significantly higher levels of amotivation towards PE. Similar results have been described in studies associating unhealthy dietary habits with reduced engagement in PA and a less favorable perception of the educational and sport context [47,48]. This finding is particularly relevant from an educational perspective, as amotivation has been linked to lower participation, reduced effort, and an increased risk of disengagement from PA.

These findings highlight the importance of promoting healthy dietary habits from early ages, not only because of their physical benefits but also due to their potential influence on key psychological variables associated with PA adherence, although the observed effect sizes were small to moderate.

On the other hand, no significant differences were observed in the dimensions of motivation towards PE according to residential environment in the present study. This finding is consistent with previous research conducted in Spanish adolescent populations, in which urban or rural environment did not show a determining effect on motivation towards PE when healthy lifestyle habits were considered as the main variables [49]. Similar patterns were identified in both urban and rural environments regarding the relationship between MD, PA, and motivation.

Notably, the lack of significant differences between rural and urban adolescents aligns with recent studies suggesting a progressive homogenization of lifestyles across both populations [50,51]. Traditionally, rural environments have been considered to promote higher levels of PA due to more frequent contact with the natural environment; however, social changes and increasing digitalization among young populations may be reducing these contextual differences [52].

This result suggests that, at least in the sample analyzed, residential environment does not constitute a determining factor in motivation towards PE, and it reinforces the relevance of healthy lifestyle habits over contextual variables.

## 5. Practical Implications

From an applied perspective, the study results highlight the need to address the promotion of PA and healthy eating in an integrated manner within the school context. Educational programmes that promote MD adherence could indirectly contribute to enhancing motivation towards PE, fostering greater student engagement in PA.

Likewise, PE teachers could play a key role in promoting healthy lifestyles by integrating content related to nutrition and well-being into their pedagogical interventions.

In this context, the results of the present study highlight the relevance of interdisciplinary approaches within educational and health settings for the promotion of healthy lifestyles during adolescence. Collaboration among different members of the educational team, including teachers, psychology professionals, and nursing staff, could contribute to more comprehensive student care, supporting not only academic development but also the maintenance of adequate physical and mental health. This joint approach would allow for a more effective response to students’ needs, promoting their well-being from a holistic perspective that integrates education in healthy dietary habits, regular PA, and the care of emotional well-being.

## 6. Study Limitations and Future Directions

This study presents several limitations that should be considered. First, its cross-sectional design prevents the establishment of causal relationships between the variables analyzed. Second, the use of self-reported questionnaires may entail social desirability bias. Third, the absence of anthropometric data (e.g., height and weight) is a limitation of the study, as body composition could act as a potential confounding factor in the relationship between diet, PA, and motivation. Future longitudinal studies would allow examination of the evolution of these behaviours and their impact on motivation over time.

Additionally, future research could include variables such as the perceived motivational climate in PE classes or teacher-provided autonomy support to gain deeper insight into the mechanisms explaining the relationship between healthy habits and motivation.

In conclusion, the results of the present study indicate that greater MD adherence and higher PA levels are associated with more self-determined motivational profiles toward PE in Spanish adolescent girls. Specifically, optimal adherence to the MD and active lifestyles are linked to higher intrinsic motivation and more adaptive forms of motivational regulation. These findings reinforce the importance of jointly promoting healthy lifestyles, highlighting the potential of the school context as a key setting for intervention.

## 7. Conclusions

The findings of this study indicate that greater adherence to the MD and higher levels of PA are associated with more self-determined motivational profiles toward PE among adolescent girls. Specifically, girls with optimal adherence to the MD exhibited higher intrinsic motivation and more adaptive forms of regulation across both residential contexts, highlighting the relevance of lifestyle behaviors in shaping motivational processes during early adolescence.

Although no statistically significant differences were observed between rural and urban participants, the consideration of contextual factors remains relevant when designing health promotion strategies tailored to adolescent populations.

These results underscore the importance of promoting healthy dietary habits and regular PA through integrated, school-based strategies that consider both nutritional education and active lifestyle promotion. Given the influential role of motivation in sustained engagement in PA, interventions aimed at improving adherence to the MD may indirectly contribute to enhancing motivation toward PE classes.

From an educational and health perspective, the findings support the need for interdisciplinary approaches involving educators and health professionals, including school nurses, to foster environments that encourage healthy behaviors and psychological well-being among adolescent girls. Future research should employ longitudinal designs to explore causal relationships and examine the long-term impact of combined lifestyle interventions on motivation and PA behaviors.

## Figures and Tables

**Table 1:** Distribution of physical activity levels and adherence to the Mediterranean diet according to the place of residence in adolescent girls.

		Urban	Rural	*p*	
		n = 108	n = 109		
Categorical variables (χ^2^)		%	%		
PA	Low	16.6	17.5	0.705	—
	Intermediate	25.8	27.2		
	High	7.4	5.5		
MD (high quality)	Low	6.9	6.9	0.94	—
	Intermediate	27.8	27.3		
	Optimal	14.2	16.2		
Continuous variables (t Student)		M (SD)	M (SD)	*p*	F
Motivation	Intrinsic	3.66 ± 0.97	3.45 ± 1.12	0.143	2.162
	Identified	3.58 ± 1.03	3.67 ± 1.01	0.545	0.368
	Introjected	3.23 ± 0.98	3.32 ± 0.90	0.464	0.539
	External	2.97 ± 1.01	3.20 ± 0.94	0.094	2.83
	Amotivation	2.48 ± 1.16	2.36 ± 1.05	0.431	0.623

Data are expressed as n (%). Differences between urban and rural environments were analysed using the chi-square test for categorical variables and Student’s *t*-test for continuous variables. *p* < 0.05 was considered statistically significant. PA: physical activity; MD: Mediterranean diet.

**Table 2:** Differences in motivation toward physical education according to the level of adherence to the Mediterranean diet in adolescent girls.

PEMQ		Descriptive Statistics					
		Mean	SD	F	*p*	η^2^p	(Post Hoc)
Intrinsic	MD Low (L)	3.27	1.11	4.14	0.017	0.037	O vs. L = 0.012 I vs. L= 0.410
	MD Intermediate (I)	3.52	1.02				
	MD Optimal (O)	3.72	1.06				
Identified	MD Low (L)	3.64	0.98	3.840	0.023	0.035	O vs. L = 0.018 I vs. L = 1.00
	MD Intermediate (I)	3.56	1.00				
	MD Optimal (O)	3.73	1.06				
Introjected	MD Low (L)	3.22	1.01	4.420	0.013	0.040	I vs. L = 1.00 O vs. L = 0.020
	MD Intermediate (I)	3.22	0.93				
	MD Optimal (O)	3.41	0.91				
External	MD Low (L)	3	0.93	0.426	0.653	0.002	—
	MD Intermediate (I)	3.07	0.93				
	MD Optimal (O)	3.18	1.06				
Amotivation	MD Low (L)	2.48	1.12	3.120	0.046	0.029	I vs. L= 0.980 L vs. O = 0.039
	MD Intermediate (I)	2.42	1.08				
	MD Optimal (O)	2.34	1.15				

Note: MD = Mediterranean diet; L = Low adherence; I = Intermediate adherence; O = Optimal adherence. Post hoc comparisons were performed using Bonferroni adjustment. Results are expressed as mean ± standard deviation (SD). Effect size was calculated using partial eta squared (η^2^p). *p* < 0.05 was considered statistically significant.

## Data Availability

The data presented in this study are available on request from the corresponding author. The data are not publicly available due to privacy restrictions.
